# Revisiting Dietary Iron Requirement and Deficiency in Indian Women: Implications for Food Iron Fortification and Supplementation

**DOI:** 10.1093/jn/nxy283

**Published:** 2019-02-09

**Authors:** Santu Ghosh, Srishti Sinha, Tinku Thomas, Harshpal S Sachdev, Anura V Kurpad

**Affiliations:** 1 Department of Biostatistics; 2 Department of Physiology, St John's Medical College; 3 Division of Nutrition, St John's Research Institute, St. John's National Academy of Health Sciences, Bangalore, India; 4 Sitaram Bhartia Institute of Science and Research, New Delhi, India

**Keywords:** iron requirement, EAR, TUL, risk of deficiency, risk of excess

## Abstract

Anemia in Indian women continues to be highly prevalent, and is thought to be due to low dietary iron content. The high risk of dietary iron deficiency is based on the Indian Council of Medical Research recommendation of 21 mg/d, but there is a need for a secure and transparent determination of the Estimated Average Requirement (EAR) of iron in this population. In nonpregnant, nonlactating women of reproductive age (WRA), the EAR of iron was determined to be 15 mg/d. Applying this value to daily iron intakes among WRA in nationally representative Indian state–based data showed that the median risk of dietary iron deficiency was lower than previously thought (65%; IQR: 48–78%), with considerable heterogeneity between states (range: 25–93%). However, in a validation, this risk matched the risk of iron deficiency as defined by blood biomarkers in a recently completed survey. When the risk of dietary iron deficiency was modelled for an increase in iron intake through food fortification of a single dietary staple, that provided 10 mg/d, the median risk reduced substantially (from 65% to 20%), and it virtually disappeared when supplementary iron intakes through the national iron supplementation program were considered. The risk of exceeding the tolerable upper level (TUL) of intake of iron remains low in the population when receiving fortification of 10 mg/d, but is much higher if they consume greater amounts of iron through supplements (range: 0–54%). This newly and transparently defined Indian EAR of iron should be used to evaluate, with precision, the benefits and risks of iron fortification and supplementation policies.

## Introduction

The National Family Health Survey, which was conducted in 1992–93 (1) and 2015–16 (2), indicates a high and continuing prevalence of anemia among children and women of reproductive age (WRA) in India. It is widely thought that the primary reason for this is an iron-deficient diet. This is because the mean reported iron density (amount of iron per 1000 kcal) in Indian diets is ∼25% lower than the recommended adequate dietary iron density of 14.2 mg/1000 kcal (3). The reported per capita median daily intake of iron in India is 14 mg/d, but is heterogeneous, ranging from 7 to 21 mg/d in different states (4). Although this is higher than the median intake of iron among WRA (12 mg/d) in the United States (5), the risk of a deficient iron intake is assumed to be profound among WRA in India, because it is much lower than the RDA of 21 mg/d that has been recommended for them (3). Therefore, enthusiastic efforts are now being made to increase the iron intake of the population through supplementation and fortification (6). However, the risk of an inadequate iron intake in vulnerable populations, for example WRA, has not been explicitly estimated in India. Defining the risk of an inadequate dietary intake requires an Estimated Average Requirement (EAR) of iron and the application of this value to the distribution of iron intake in the population under consideration, in what is called the EAR cut-point or the probability method (7). However, the daily iron requirement in India is currently available only as RDA, which is meant to define the requirement of an individual where an intake equal to this would put the individual at a very small (2.5%) risk of an inadequate intake, suggesting that the individual is likely to be meeting their individual requirements; however, since the converse is not true, the RDA is not considered to be a useful reference standard for assessing the adequacy of nutrient intakes (7). The RDA should not be used for estimating the risk of inadequate intakes in populations. At the other end of the spectrum of nutrient intakes, the safe level of intake, beyond which the risk of adverse events begins to increase, is called the Tolerable Upper Level (TUL) of intake (7). This value, which is the sum of all sources of nutrient intake, including supplements, acts as a guideline of safety to help ensure that nutrient intakes do not habitually exceed this value. For the iron intake of WRA, the TUL value is currently set at 45 mg/d (8). A recent Institute of Medicine/WHO/FAO workshop on nutrient intake recommendations stated that transparent and rigorous determinations of nutrient requirements were essential for accurate policy formulation (9). If policies to increase iron intake through fortification and supplementation are not informed about the true risk of inadequate dietary intake, they could fail.

## Revisiting the Daily Iron Requirement in Indian WRA

The mean daily physiological requirement of iron is calculated by a factorial method, which sums components of daily iron loss from the body. For non-pregnant, non-lactating WRA, these components are the daily basal iron loss and menstrual loss of iron. This summation uses the mean value for each factor and yields the daily estimated mean physiological requirement for iron. The EAR is then derived as the ratio of the physiological requirement to the bioavailability of iron from the diet. The RDA, which is defined as the EAR plus 2 SDs of the distribution of requirements, is also adjusted for bioavailability. This is the value at which the risk of inadequate dietary iron intake in an individual is <2.5% (7). The present Indian recommendation (3) derived the daily iron requirement for WRA using this factorial method; however, because this value was derived as the sum of the mean basal loss and the 97.5th percentile of the menstrual loss, it was neither an EAR nor an RDA.

To estimate the EAR and RDA of the iron requirement of WRA within their strict definitions, first, the distribution of the basal loss of iron was required. Since no data were available from India, data reported in adult males from Seattle, Venezuela, and South African Indians were used, because the difference in measured loss between these groups was not significant (10). The mean basal iron loss relative to body weight was 14 µg · kg body weight^–1^ · d^–1^ with a CV of 29.2%. To estimate variability in basal loss in WRA, the CV of basal loss per kilogram of body weight was combined with the mean CV of body weight in WRA between 18 and 49 y of age, which was estimated to be 15.6% from national anthropometric data (3), as the variance of the product of two independent random variables. The reference body weight of WRA was assumed to be 55 kg (3), yielding a mean ± SD daily iron loss of 0.77 ± 0.25 mg/d. The probability distribution of basal loss was assumed to be normal.

**FIGURE 1:**
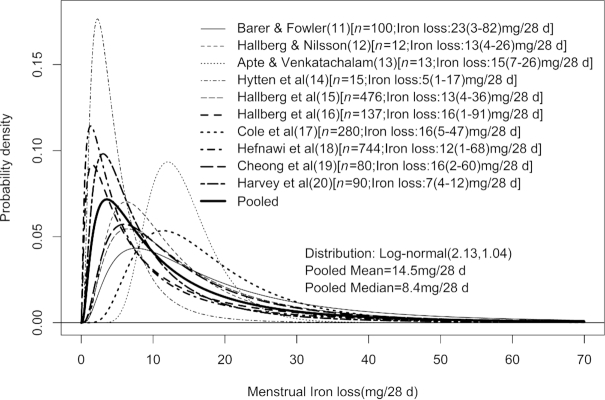
Estimated distribution of menstrual iron loss derived from different studies. The values within square brackets represent the sample size of the study and estimated mean iron loss (minimum loss − maximum loss). Log-normal (2.13, 1.04): Log-normal distribution with mean at log-scale = 2.13 and SD at log-scale = 1.04.

Second, for the distribution of iron losses due to menstruation, a search for related literature was conducted in PubMed, with search terms of “iron loss,” “menstrual blood loss,” “menstrual iron loss,” and “women in reproductive age.” Studies on women aged <15 or >50 y, severely anemic women, lactating women, and on those using intrauterine devices or oral contraceptives were excluded, yielding 10 studies (11–20). Only 5 reported iron loss, whereas the rest reported blood loss. Where blood loss was reported, the iron loss was derived as the product of the daily blood loss assuming a mean of 28 d in a cycle (21), the hemoglobin (Hb) concentration taken as 135 g/L unless otherwise reported (5), and the iron content of Hb taken as 3.39 mg/g (22). In a validation of this approach, within the studies that reported iron loss, the mean bias of the calculated iron loss was only 0.05 mg/d. Because the distribution of the menstrual iron loss appeared to be positively skewed in many studies, its probability distribution was taken as lognormal. The mean and variance at log scale, as µ and σ^2^, were estimated from the reported dispersion for each study separately. The reported range was assumed to be from the 2.5th percentile (min, L_p_) to the 97.5th percentile (max, U_q_). µ and σ^2^ were estimated for each study from ${{\Phi }}({\frac{{\log ({{L_p}}) - \mu }}{\sigma }} ) = \ p\ \& \ {{\Phi \ }}( {\frac{{\log ( {{U_q}} ) - \mu }}{\sigma }} ) = \ q$; where $\Phi(.)$ is the cumulative distribution function of the standard normal distribution, and L_p_ and U_q_ are the p^th^ and q^th^ percentiles, respectively. The pooled estimates of µ and σ were finally derived as the weighted (based on the sample size) mean of the estimates obtained from all the studies (**Figure 1**).

Third, the absorption of dietary iron from different cereal-based Indian meals was determined from published reports on Indian WRA and adolescent girls (**Table 1**) (23–26). The iron absorption from different cereal-based Indian meals was considered as the National Sample Survey 68th round showed that nearly 70% of the iron consumed is from cereals and only about 1% from heme sources (27). These were based on a search of PubMed using the search terms “iron absorption,” “stable isotope,” and “India” which yielded 6 articles, of which 4 were selected, which used accurate iron absorption methods, which measured the incorporation of a stable isotope of iron provided in a common pool of iron from a meal into Hb, and reported the mean and SD of iron absorption in anemic and normal WRA. A mean absorption was calculated using the weighted inverse of the SE of each study, the proportion of anemic and normal WRA in survey data (2), and the proportion of consumption in weight of rice, wheat, and millet in the total cereal intake in the Indian population (4, 27). The mean absorption of dietary iron was 8.7% which is similar to the value of 8% used for WRA in the current recommendation (3), and this latter value was used to adjust the physiological requirement of iron to obtain the EAR.

**TABLE 1:** Reported iron absorption among WRA and adolescent girls in India^1^

Study	Age (y)	Sample	Anemia status	Cereal source	Meal iron (mg)	Isotope iron (mg)	Absorption^2^ (%)
Thankachan et al. (23)	18–35	20	Anemic	Rice	1.3	3.0	17.5 ± 11.4
	18–35	20	Normal	Rice	1.3	3.0	7.3 ± 5.9
Kalasuramath et al. (24)	18–35	15	Anemic	Rice	2.5	3.0	8.3 ± 2.2
	18–35	15	Anemic	Wheat	3.4	3.0	11.2 ± 1.6
	18–35	15	Anemic	Ragi (millet)	2.7	3.0	4.6 ± 1.9
	18–35	15	Normal	Rice	2.5	3.0	2.7 ± 1.7
Herter-Aeberli et al. (25)	18–35	16	Normal^3^	Rice	1.3	5.0	10.0 ± 6.5
	18–35	13	Normal	Rice	1.3	5.0	16.7 ± 4.6
Nair et al. (26)	13–15	16	Normal	Rice	10.8	3.4	9.7 ± 6.5

^1^All studies followed a stable isotope iron absorption method to measure the absoprtion of iron from the meal.

^2^Values are means ± SDs.

^3^Overweight.

**FIGURE 2:**
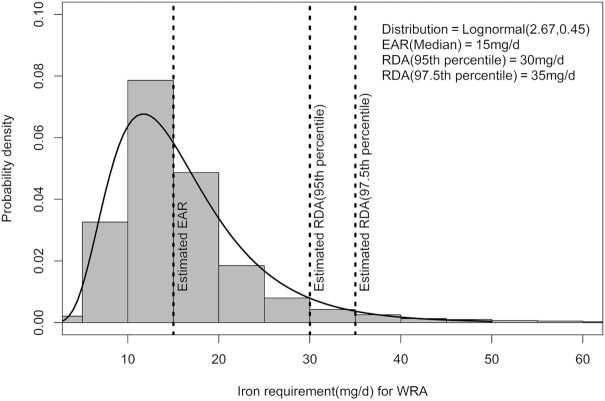
Current estimate of EAR and RDA of iron per day for Indian WRA. Log-normal (2.67, 0.45): log-normal distribution with mean at log-scale = 2.67 and SD at log-scale = 0.45. EAR, Estimated Average Requirement; WRA, Women of Reproductive Age.

The distribution of iron requirements was obtained by convolution of the probability distribution of daily basal and menstrual iron loss. Because no close form of the convolution of lognormal and normal distribution exists, Monte Carlo simulations were performed to obtain an approximated distribution of iron requirement. Finally, the median and 95th or 97.5th percentile were derived from estimated iron requirement distributions to represent the physiological EAR and RDA. These values were corrected for a dietary iron absorption of 8%, to yield an EAR of 14.4 mg/d (rounded off to 15 mg/d) and RDA of 30 mg/d or 35 mg/d for the 95th or 97.5th percentile, respectively (**Figure 2**). The RDA is high because the CV of the basal loss (which was substantial) was also considered in the calculation of the total variability of iron loss; this variability was not considered in earlier estimations of the RDA.

There are some limitations in the factorial method as used here. Because data were not available for WRA, it was assumed to be similar to the basal iron loss reported for adult men. This is not unreasonable, as there is no reason to infer that these losses are different between sexes. From the extracted literature, only one study on menstrual blood loss was conducted on Indian WRA, but owing to these limited data, all relevant studies were included in the analysis. Finally, it is worth pointing out that an additional loss of iron could occur owing to helminth infections, and the prevalence of soil-transmitted helminth infections is high in some states in India (28). However, this was not considered in the present computation because the requirement for iron is derived for healthy WRA, there is considerable heterogeneity in the prevalence of parasitic infections, and the effect of deworming on Hb is equivocal (29). It is also difficult to estimate the potential blood loss in this condition with any accuracy for a factorial method.

## Revised Risk of Inadequate Iron Intake

To evaluate the risk of an inadequate iron intake in the Indian population, nationally representative survey data on daily iron intake (both heme and nonheme sources) for each Indian state and union territory were obtained from the National Sample Survey Office on household expenditure (27). Monthly per capita consumer expenditure as well as the household food purchase of 223 food items were collected through this survey, with a recall period of 30 d. The quantities of different foods purchased by a household were converted to nutrients of interest using the Indian food composition tables (30), and adjusted for the number of members in the household, to obtain the daily per capita iron intake. The maximum likelihood estimation technique was applied to estimate the appropriate parametric distribution of usual intake of dietary iron for each state and union territory. The distribution of population risk of inadequate intake of iron was derived by using the probability approach (7), along with either the EAR in this report (15 mg/d) or the current Indian requirement value of 21 mg/d (3). The mean of the risk function $\rho (x) = 1 - {\rm F}({\rm x})$; where F(x) is the cumulative distribution function of the estimated requirement distribution (Figure 2), evaluated for a large number of random samples simulated from estimated usual intake distribution by the Monte Carlo approximation method (31), was the estimate of the WRA population at risk of inadequate iron intake. The median risk of inadequate intake of iron in all Indian states and union territories, estimated with the current report's EAR, was ∼28% lower, ranging from 6% to 50% of the risk derived using the present Indian recommendation (**Table 2**). The IQR of the risk lay between ∼48% and 78% compared to the earlier 79–97%.

**TABLE 2:** Estimates of the risk of inadequate or excess iron intake before and after iron fortification and supplementation in the states and union territories of India^1^

	Risk of inadequate intake of iron in habitual diets		Risk of inadequate intake after iron fortification and supplementation		Risk of excess intake after iron fortification and supplementation	
State	EAR: ICMR 2010^2^	EAR: Estimated^3^	10 mg/d^4^	24 mg/d^5^	10 mg/d^6^	24 mg/d^7^
A&N Island	81	51	14	2	0	12
Andhra Pradesh	97	78	26	3	0	0
Arunachal Pradesh	95	80	33	5	0	3
Assam	98	83	33	5	0	0
Bihar	82	49	13	2	0	7
Chandigarh	78	48	20	3	0	14
Chhattisgarh	97	78	31	4	0	1
D&N Haveli	92	73	28	4	0	5
Daman & Diu	83	50	13	2	0	5
Delhi	81	49	13	2	0	8
Goa	95	72	28	4	0	1
Gujarat	76	45	13	2	0	16
Haryana	65	33	8	1	0	27
Himachal Pradesh	66	33	8	1	0	24
Jammu & Kashmir	87	57	16	2	0	4
Jharkhand	90	65	20	3	0	4
Karnataka	89	60	17	2	0	3
Kerala	94	70	22	3	0	1
Lakshadweep	86	56	16	2	0	6
Madhya Pradesh	66	36	9	1	1	29
Maharashtra	79	48	11	1	0	12
Manipur	100	93	37	5	0	0
Meghalaya	100	91	39	5	0	0
Mizoram	98	83	34	5	0	0
Nagaland	99	87	32	4	0	0
Orissa	97	78	25	3	0	0
Puducherry	94	68	20	3	0	0
Punjab	66	34	8	1	0	25
Rajasthan	49	25	6	1	1	54
Sikkim	99	83	32	5	0	0
Tamil Nadu	98	79	26	3	0	0
Tripura	98	77	30	4	0	0
Uttar Pradesh	75	42	11	1	0	15
Uttaranchal	69	36	9	1	0	20
West Bengal	93	68	26	4	0	2

^1^Values are percentages. A&N Island, Andaman and Nicobar Islands; D&N Haveli, Dadar and Nagar Haveli; EAR, Estimated Average Requirement; ICMR, Indian Council of Medical Research.

^2^EAR value of 21 mg/d (3).

^3^Current estimate of EAR value: 15 mg/d.

^4^Risk of inadequate iron intake with estimated value of EAR 15 mg/d after iron fortification (10 mg/d).

^5^Risk of inadequate iron intake with estimated value of EAR 15 mg/d after iron fortification (10 mg/d) and supplementation (14 mg/d). The value of 24 mg is the sum of 10 and 14 mg/d. The latter is assumed to come from a weekly dose of 100 mg elemental iron in supplementation programs.

^6^Risk of excess iron intake over a TUL of 45 mg/d after iron fortification (10 mg/d).

^7^Risk of excess iron intake over a TUL of 45 mg/d after iron fortification (10 mg/d) and supplementation (14 mg/d). The value of 24 mg is the sum of 10 and 14 mg/d. The latter is assumed to come from a weekly dose of 100 mg elemental iron in supplementation programs.

The validity of these new estimates of risk of inadequacy was tested by comparing them with biomarker-based (α-glycoprotein and C-reactive protein–adjusted serum ferritin) measurements of iron deficiency in WRA, where available. In a recently conducted survey in one-third of the districts in Uttar Pradesh (32), the prevalence of iron deficiency was 51% in WRA, which compared well with the present new estimate of the risk of dietary iron inadequacy (42%) in all districts in Uttar Pradesh (Table 2).

Based on the EAR proposed here, the prevalence of risk of dietary iron inadequacy in WRA is much lower than previously thought. One might expect to reduce this risk further (because the requirements are positively skewed) through food fortification, which could deliver ∼10 mg/d if a single food staple were fortified. With this single-food fortification policy, the risk of iron inadequacy would now range from 6% to 39% in different states, with a median risk of 20% (Table 2). If a further additional 14 mg iron/d were to be provided by programmatic iron supplementation through the National Iron Plus Initiative (providing 100 mg/wk to WRA) (5), the risk of inadequacy would virtually disappear to 1–5% in different states, with a median risk of 3%. However, it is important to emphasize that a new risk, of exceeding the TUL of iron intake (45 mg/d), can appear to a significant extent, to as high as 54% in some states, when iron is supplied through both fortification and supplementation (Table 2). This demands a precision-based approach, entailing more information on iron absorption from different diets, and a careful reappraisal of the risks and benefits of increasing iron in the diet through supplementation and fortification needs to be performed in India, such that benefits can be maximized at the lowest risk.

## Notes

Supported by the Margdarshi Fellowship from the India Alliance of the Wellcome Trust and the Department of Biotechnology (to AVK).

Author disclosures: SG, SS, TT, HSS, and AVK, no conflicts of interest.

Abbreviations used: EAR, Estimated Average Requirement; Hb, hemoglobin; TUL, Tolerable Upper Level; WRA, women of reproductive age.
